# From eligibility to diagnosis: candidacy and the complex journey of cerebral palsy diagnosis within primary care

**DOI:** 10.1186/s12887-025-05455-5

**Published:** 2025-02-12

**Authors:** Jessica Baggaley, Charlotte Seiboth, Tim Rapley, Anna Basu

**Affiliations:** 1 Population Health Sciences Institute, Newcastle University, Newcastle upon Tyne, UK; 2 Social Work, Education and Community Wellbeing, Northumbria University, Newcastle upon Tyne, UK; 3 Paediatric Neurology, Great North Children’s Hospital, Newcastle upon Tyne, UK

**Keywords:** Cerebral palsy, Primary care, Diagnosis, Early diagnosis, Survey, Interview, Infant, Candidacy, Referral

## Abstract

**Background:**

Cerebral Palsy (CP) is an umbrella term for a group of permanent postural and movement conditions caused by non-progressive damage to the developing brain. Infants not identified with risk factors for CP around the time of birth are usually referred on from primary care after six months of age, essentially precluding early therapy. Candidacy, a seven-step dynamic theory, describes how individuals negotiate their eligibility for medical attention with themselves, others, and health services. This study aims to explore the CP diagnostic journey for community identified infants using the concept of candidacy.

**Methods:**

Data was combined from two studies: an online survey of caregivers of children with CP about their earliest concerns and diagnosis journeys (*n=*255), and a series of interviews to support the development of a new tool to facilitate earlier identification of infants with emerging motor difficulties (11 parents, 11 health care professionals [HCPs]). A deductive thematic analysis was used with a semantic, critical realist approach. An initial analysis was framed by the Andersen Model of Total Patient delay, and then conceptualised using Candidacy.

**Results:**

Participants had difficulties identifying whether their child needed medical attention, prompting online searches, and seeking advice from family and friends. HCP adjudications led to immediate or delayed referral, in which families continued their searches, reappearing at services until a referral was made. Once referred, families faced poor operating conditions, such as long waiting times. After learning the diagnosis criteria, participants began making requests for referral and navigated to private services if requests were denied. Participants felt that more information on infant development from a reliable source was needed to support new parents in raising their concerns to aid earlier identification.

**Conclusion:**

Participants identified personal lack of infant development knowledge as being the limiting factor to earlier referral. Further research is needed to develop materials relevant for the UK and to understand General Practitioner perspectives regarding provision of such materials.

## Introduction

### Identifying cerebral palsy in the UK

Cerebral Palsy (CP) is an umbrella term for a group of permanent posture and movement disorders due to non-progressive damage to the developing brain, often accompanied by associated impairments and secondary musculoskeletal problems [1]. It is also the most common childhood disability, with a worldwide prevalence of 2.1 per 1000 births [2] and a prevalence of 1.6 per 1000 live births in high income countries [3].

Research has shown that early intervention (0–2 years) in CP has significant positive impacts on motor and cognitive outcomes [4]. Recommendations on early intervention for this group are aimed at infants with motor dysfunction and either abnormal neuroimaging or clinical history that indicates high risk for CP [4].

In the UK infants with identifiable major risk factors for CP receive clinical follow up by a multidisciplinary team between birth and 2 years of age. These infants also receive standard developmental screening, carried out by Health Visitors. Standard developmental screening consists of postnatal visits at 5–8 days, 10–14 days, 6–8 weeks, 9–12 months and 2-2½ years of age, with the first standardised assessment of infant development occurring at the 9–12 month visit [5].

However, around 40–50% of infants with CP do not demonstrate risk factors for CP at around the time of birth [6]. These ‘low-risk’ infants only receive standard developmental screening, and thus rely on their caregivers and primary health care professionals (PHCPs) identifying and subsequently reporting their earliest signs of CP, resulting in a referral to secondary care for diagnosis and therapy. In Canada, low-risk infants have been demonstrated to be referred to secondary care on average later (28.8 months± 27.1) than the 2-years-of-age cut off which defines early therapy [7].

### Candidacy

Candidacy describes how an individual’s eligibility for health care is negotiated within themselves, with others, medical staff and health care services and is constantly defined and redefined through their interactions [8]. It accounts for the work the user puts in, as well as the amount, difficulty and complexity of that work that may act as barriers to accessing care. It encompasses social patterning of perceptions of health and health services and the alignments between priorities and competencies of the individual and the health services, and it describes the availability of resources, such as time and policy. Candidacy is made up of seven stages. Stage 1 (identification of candidacy) describes the process in which an individual comes to recognise their symptoms as needing medical attention [8]. Stage 2 (navigation of services) describes an individual’s knowledge of the services provided and understanding on how to make contact with and how to access services [8]. Stage 3 (permeability of services) describes the ease with which an individual can access services [8]. Services are classified by their permeability, with porous services requiring fewer candidacy qualifications to access, for example Accident and Emergency, whereas low permeability services, such as referral to secondary care, demand candidacy qualifications. Stage 4 (appearance at services) describes the individual’s ability to assert their candidacy for medical care [8]. To make a claim, individuals need to be able to formulate and articulate their issues credibly. Stage 5 (adjudication by Health Care Professionals (HCPs), describes how an individual is judged by their HCPs, and how that judgement subsequently influences their progression through the services and their access to care [8]. Ultimately adjudication results in an individual being classified as being deserving or not deserving of care. Stage 6 (offers and resistance to services) describes how an individual may refuse offers made to them by HPCs at multiple stages of their journey, including resisting appointment, referral, and treatment offers [8]. Stage 7 (operating conditions and local production of candidacy) describes the social and macro level factors that influence candidacy [8]. Examples of these factors include the availability of local resources and the relationship that develops between the HCP and the individuals over multiple visits.

These seven stages are dynamic, meaning that the stages may not occur in a linear order, and that individuals may experience multiple stages of candidacy at once, or jump forwards and backwards between stages based on the interactions they have.

Understanding the low-risk CP journey through the lens of candidacy may demonstrate areas in which intervention could be targeted to reduce the length of time to referral. To our knowledge there is no literature looking at the diagnosis journey of these low-risk infants. Therefore, this study aims to understand the diagnostic journey caregivers experience when their infant’s emerging CP is identified in the community, explore the delays caregivers experience using the candidacy framework, and what could be done to reduce these delays, predominantly based on the caregiver perspective, supplemented with primary and secondary health care professional perspectives.

## Methods

The data in this report comes from two interrelated studies: an online survey of UK caregivers of children with CP about their earliest concerns and the diagnosis pathway and a series of interviews with key stakeholders to discuss the development of a new tool aimed at supporting earlier identification and referral of infants with emerging movement problems. Key stakeholders included parents of children with typically developing children, parents of children diagnosed with CP, general practitioners, health visitors, conductive education teachers, paediatric physiotherapists, a paediatric speech and language therapist, and a paediatric occupational therapist. Ethical approval was granted by Newcastle University’s Research Ethics Committee for the online survey (1677/9309/2018). Wales REC 7 granted ethical approval for the interviews (19/WA/0328).

### Online survey

#### Study design

The design was a qualitative research design using a cross sectional online questionnaire. A critical realist approach (post positivistic philosophy) was used to to allow for reductive reasoning, in which participant experiences are developed into a single narrative. This was so that delays that find their origin within the candidacy stages, that caregivers commonly experience, could more clearly be identified as targets for intervention.

#### Participants

Participants were required to be a parent or caregiver of a child with CP, to provide informed consent, and show an ability and willingness to complete the survey. Participants were excluded if they did not look after the child in question before the child received a diagnosis of CP, they responded about a child who did not have a formal diagnosis of CP, or if they resided outside of the UK.

#### Materials

The survey consisted of items covering the child’s demographics, the earliest concerns caregivers had regarding their child’s development, who reported the concerns, to whom the concerns were reported, the caregiver’s experience of the referral and diagnosis process, and caregiver demographics. The survey was made up of multiple choice and free text items. Free text items were used for topics such as earliest concerns and experiences to reduce bias. All questions were forced response; however, participants were made aware that they could respond with ‘N/A’ if they did not wish to answer the question.

The Gross Motor Function Classification System Family Report Questionnaire (GMFCS) [9, 10] was included to assess the severity of the child’s CP. The GMFCS is a five-level ordinal parental-report classification system for describing the mobility of a child with CP. A score of 1 indicates limited mobility. A score of 5 indicates severe limitations on mobility such as requiring a wheelchair and physical assistance. The GMFCS is validated for children aged 2–18 years. Caregivers of children <2 years of age were not given the GMFCS. Caregivers whose children were 18+ years were given the 12 to 18 years questionnaire as it has been shown to be reliable in adults [11].

The survey was reviewed by the UK charity Scope (scope.org.uk) and by 22 individuals known to the team from a variety of educational levels (in order of pilot testing, 6 researchers, 3 clinicians, 7 postgraduates, 3 undergraduates, and 3 college educated students). The survey was then piloted within 3 parents of children with CP known to the team. Both stages of pilot testing were carried out in an iterative manner until 2 testers raised no additional comments.

#### Procedure

Participants were recruited to the survey using e-flyers through UK based charities: Bobath (bobath.org.uk), Cerebra (cerebra.org.uk), CP UK (cerebralpalsy.org.uk), Heel & Toe (heelandtoe.org.uk) and Scope (scope.org.uk), Parent Carer forums and through social media posting. Participants were asked to share the survey link with their friends and followers on social media to encourage snowball sampling [12–14].

The survey was hosted online using Boston Online Surveys (https://www.onlinesurveys.ac.uk/, Jisc, Bristol, UK). Participants went through the survey items in the order described in Materials.

The survey was open between 5/6/2019 and 15/11/2019. In that time the survey was accessed 2,328 times with 266 full responses given. 11 responses were excluded due to the participant not being UK based (*N*=4), likely erroneous reported limb involvement distribution, such as just the neck being affected (*N*=4), and no information given about the earliest concerns they had (*N*=3).

#### Qualitative data analysis

All qualitative analysis was carried out in NVivo 12 (Version 12.6.0.959; QSR International) after pseudonymisation.

This study used a deductive framework thematic analysis as described by Braun and Clarke [15]. Initially the data was coded in line with the Andersen model of total patient delay [16]. The Andersen model was originally developed for identifying delays in the cancer diagnosis pathway. It splits the patient journey from the identification of the first symptom to receiving treatment into 4 stages: ‘Appraisal’ (the time between identifying a symptom and seeking help), ‘Help-seeking’ (the time between deciding to seek help and the first appointment with a HCP), ‘Diagnosis’ (time between first appointment and receiving a diagnosis), and ‘Pre-treatment’ (time between receiving a diagnosis and beginning treatment). Once the data was coded to the Andersen model, thematic analysis was undertaken using the seven stages of the Candidacy model [8]. In both steps the data was analysed at a semantic level to ensure the researchers did not add additional meaning to the participant’s words.

Andersen model coding was carried out by JB and CS. Candidacy coding and theme development was carried out by JB and was reviewed by TR and AB. JB developed the survey and interview guides as part of her PhD. Although the interviews were not focused on the patient journey JB allowed participants to speak freely about this to help inform the development of tools to improve the CP diagnostic and referral process.

At the time of analysis, CS was a Psychology Undergraduate student, and JB was undertaking a PhD exploring parent experiences of the diagnostic pathway for their child with CP. TR has a PhD in sociology, and AB is a clinical senior lecturer and consultant paediatric neurologist.

### Interviews

#### Study design

The design was a qualitative research design using in depth interviews. The interview data was analysed using a critical realist approach (post positivistic philosophy) to allow for reductive reasoning in which participant experiences are developed into a single narrative. This was so that any areas of the journey in which delays that find their origin within the candidacy stages, that caregivers commonly experience, could be identified as targets for intervention.

#### Participants

Participants were either required to be a parent or caregiver to a typically developing child or a child diagnosed with CP, or to be a HCP working in primary care (GP, Health Visitor), or in secondary care with infants and have a specialist knowledge of CP (paediatricians, occupational therapists, physiotherapists, and speech and language therapists). As the aim of the interviews were to develop a new tool to help earlier identification of infants with CP, parents of typically developing children were also invited to take part to ensure the final tool would not cause unnecessary worry and to check acceptability.

#### Materials

An initial list of features seen in emerging CP was developed based on the concerns reported in the survey. The list was presented to interviewees and was iteratively developed into information sheets throughout the interviews, based on the participant responses, through a participatory design process.

The topic guides used in the interviews were developed iteratively through meetings of the research team and consultation with outside experts. The topic guides focused on the items included, the language used, and the design of the tool, such as how it should be presented (i.e. questionnaire), and how the tool would be used.

#### Procedure

Potentially eligible parents were made aware of the interviews through their local parent carer forums and through online social media posting of survey e-flyers on Facebook and Twitter by the research team. HCPs were also approached by their governing bodies (Royal College of Occupational Therapists; The Chartered Society of Physiotherapy), by their NHS Trust (The Newcastle Upon Tyne Hospitals NHS Foundation Trust) and Primary care facilities. Participants were encouraged to share the e-flyer with anyone they thought might be interested in taking part.

The interviews were carried out over Microsoft Teams. Participants were sent information packs at least 48 hours before their interview consisting of an information sheet, a consent form, and a sociodemographic questionnaire. At the start of the interviews, participants were required to give verbal informed consent. Interviews were audio recorded and transcribed verbatim before being pseudonymised.

#### Qualitative data analysis

All qualitative analysis was carried out in NVivo 12 (Version 12.6.0.959; QSR International) after pseudonymisation.

This study used a deductive framework thematic analysis as described by Braun and Clarke [15]. Initially the data was coded in line with the Andersen model of total patient delay [16]. Once the data was coded to the Andersen model, thematic analysis was undertaken using the seven stages of Candidacy model [8]. In both steps the data was analysed at a semantic level to ensure the researchers did not add additional meaning to the participant’s words. Andersen model coding was carried out by JB and CS. Candidacy coding and theme development was carried out by JB and was reviewed by TR and AB.

## Results

### Participants

#### Survey

Two hundred fifty-five responses were analysed. 240 responses (94.1%) were from mothers and the respondent median age was 39 years (Range 20–73 years). Respondent demographics are shown in Table 1. The media age category of the children described was 6–11 years, the most frequently reported limb involvement distribution was Hemiplegia and the modal GMFCS 2 (Table 2). 56.3% participants reported concerning features within primary care. 1 child was identified by their schoolteacher who reported their concerns to secondary care. It was unclear as to where concerning features were identified for 8 children. 34.1% of the sample was diagnosed by the age of 1 year, 69.4% by the age of 2 years, and 87.1% by the age of 3 years. 1 child was diagnosed after 6 years of age.

**Table 1:** Survey respondent demographics

***Relationship to the infant***									
*Mother*			*Father*		*Grandmother*			*Other family member*	
240 (94.1%)			8 (3.1%)		6 (0.4%)			1 (0.4%)	
***Highest level of education***									
GCSE or equivalent			A Level or equivalent					University Degree	
41 (16.1%)			67 (26.3%)					147 (57.6%)	
***Ethnicity***									
*White European*		*Asian other*	*Black African*		*Black Caribbean*			*Indian*	*Other*
248 (97.3%)		1 (0.4%)	1 (0.4%)		1 (0.4%)			1 (0.4%)	3 (1.2%)
***Employment status***									
*Full time work*	*Part time* work	*Full time * *carer*	*Homemaker*	*Seeking work*	*Unemployed due to health*	*Retired*	*Full time student*	*Mat/* *Pat leave*	*Other*
65 (25.5%)	85 (33.3%)	63 (24.7%)	22 (8.6%)	2 (0.8%)	3 (1.2%)	1 (0.4%)	2 (0.8%)	4 (1.6%)	8 (3.2%)
***Marital status***									
*Married/Civil partnership/Co-habiting with long term partner*			*Divorced/Separated*		*Single*			*Widowed*	
209 (82%)			16 (6.3%)		28 (4.3%)			2 (0.8%)

**Table 2:** Children’s demographics from the survey. CP – Cerebral Palsy. GMFCS – Gross Motor Function Classification System

***CP Type***					
Hemiplegia	Quadriplegia	Diplegia	Triplegia	*Monoplegia – Lower limb*	*Monoplegia – Upper limb*
118 (46.3%)	75 (29.4%)	36 (14.1%)	20 (7.8%)	5 (2.0%)	1 (0.4%)
***Age Group***					
*Under 2 years*	*2–3 years*	*4–5 years*	*6–11 years*	*12–17 years*	*≥18 years*
17 (6.7%)	61 (23.9%)	45 (17.6%)	77 (30.2%)	34 (13.3%)	21 (8.2%)
***GMFCS***					
*Under 2 years*	1	2	3	4	5
17 (6.7%)	63 (24.7%)	67 (26.3%)	46 (18%)	27 (10.6%)	35 (13.7%)

#### Interviews

A total of 25 people expressed interest in being interviewed. Of these, one stopped responding to emails before an interview was arranged, one became ill and was unable to take part, and one secondary care HCP did not work with infants with CP. In total 22 people (50% parents) took part. Only one person (Alice; mother of a child with CP) took part in more than one interview. Information about the parents and their children, and HCPs is shown in Tables 3 and 4, respectively. 1 parent and 1 HCP did not complete sociodemographic questionnaires.

**Table 3:** Demographic information about the parent. Note: f - denotes female, m - denotes male

*Participant*	*Interview (I) number*	*Relationship to child*	*Child’s name*	*Cerebral Palsy type or TD*	*Child’s age*	*Age of TD siblings*
*Alice*	I1	Mother	Alex (m)	Hemiplegia	20 m	
*Bethany*	I1	Mother	Briar (f)	Hemiplegia	5 y	
*Claire*	I2	Mother	Cara (f)	Hemiplegia	16 y	18 y, 14 y
*Daisy*	I2	Mother	NA (m)	Quadriplegia	10 y	5 y
*Keira*	I8	Legal guardian	Kali (f)	TD	3 y	
*Nicole*	I9	Mother	Nina (f), Nathaniel (m), and Nico (m)	TD	15 m, 5 y and 7 y	
*Noah*	I9	Father	Nina (f), Nathaniel (m), and Nico (m)	TD	15 m, 5 y and 7 y	
*Petra*	I10	Mother	Perry (m)	Hemiplegia	26y	26y; 26y (triplets)
*Phineas*	I10	Father	Perry (m)	Hemiplegia	26y	26y; 26y (triplets)
*Riley*	I12	Mother	Rose (f)	Hemiplegia	2.5 y	7 m
*Violet*	I14	Mother	Vivian (f)	Hemiplegia	10 y	10 y (Twin)

**Table 4:** Demographic information, health care professionals

*Participant*	*interview (I) number*	*Profession*
*Elaine*	I3	Conductive Education teacher
*Francis*	I3	Community physiotherapist
*Grace*	I3	Conductive Education teacher
*Heather*	I4	Paediatric occupational therapist
*Iris*	I4	Paediatric occupational therapist
*Jackie*	I5	Paediatric speech and language therapist
*Kaia*	I6	Health Visitor
*Lily*	I7	Health Visitor
*Madelyn*	I7	Health Visitor
*Sam*	I13	GP
*Thomas*	I15	GP

Participants were aged between 31–61 years (*n=*19, mean age 48 years, 1 participant gave an age range of 50–60 years). The majority were White European, with a university degree (Diploma = 1; GCSE or equivalent = 1), and were married, in a civil partnership, or were cohabitating with long term partner (Widowed = 1, Prefer not to say = 1). HCPs had a mean of 28 years of experience (*n =* 9, range = 28 years); 1 participant reported ‘20+years’ of experience). Ages of the children ranged from 20 months to 26 years. All but one of the children with CP had Hemiplegia.

### Summary of findings

Caregivers’ descriptions of the diagnosis journey described 3 of the 4 stages of the Andersen model (Appraisal, Help seeking, and Diagnosis). Across these three Andersen stages, the descriptions covered 6 of the 7 candidacy stages. The order in which caregivers described the candidacy stages occurring followed the pathway shown in 1. In summary, their journeys started with initially noticing their infants’ symptoms, *identification of candidacy,* which led directly into *appearance at services* as they sought out friends and family or searched online for information and advice about their observations. Their findings directly impacted on their *navigation to services*, with some delaying seeking help due to friends or family not being concerned, or being worried that they would appear to be neurotic or paranoid. Caregivers reported being given *adjudications* for and against referral. Those who did not receive referral continued their primary care journey through continuing to build their evidence to support their *appearance at services,* and/or to *navigate* back to the same or a different PHCP. This loop continued until a referral was made. When referrals did occur, families faced further additional barriers due to low secondary care *permeability*, a lack of resources resulting in delays, and poor navigation by HCPs on the family’s behalf. However, as caregivers became more knowledgeable about the requirements for diagnosis, they themselves began to be able to produce candidacy themselves. This self-*production of candidacy* allowed them to *navigate* services, such as requesting Magnetic Resonance Imaging (MRI) brain scans and referral to specialists. When *local operating conditions* were creating barriers, such as long waiting lists, families decided to pay for MRI brain scans through private care pathways. The only stage not described by caregivers was ‘offers and resistance to services’.

Suggestions on how to improve identification of infants with CP within the community did not fit within the Andersen model stages, and so was developed into its own theme. Caregivers felt rather than additional screening, new families need more information on infant development from a reliable source to help them identify what is typical and atypical.

**Fig. 1:**
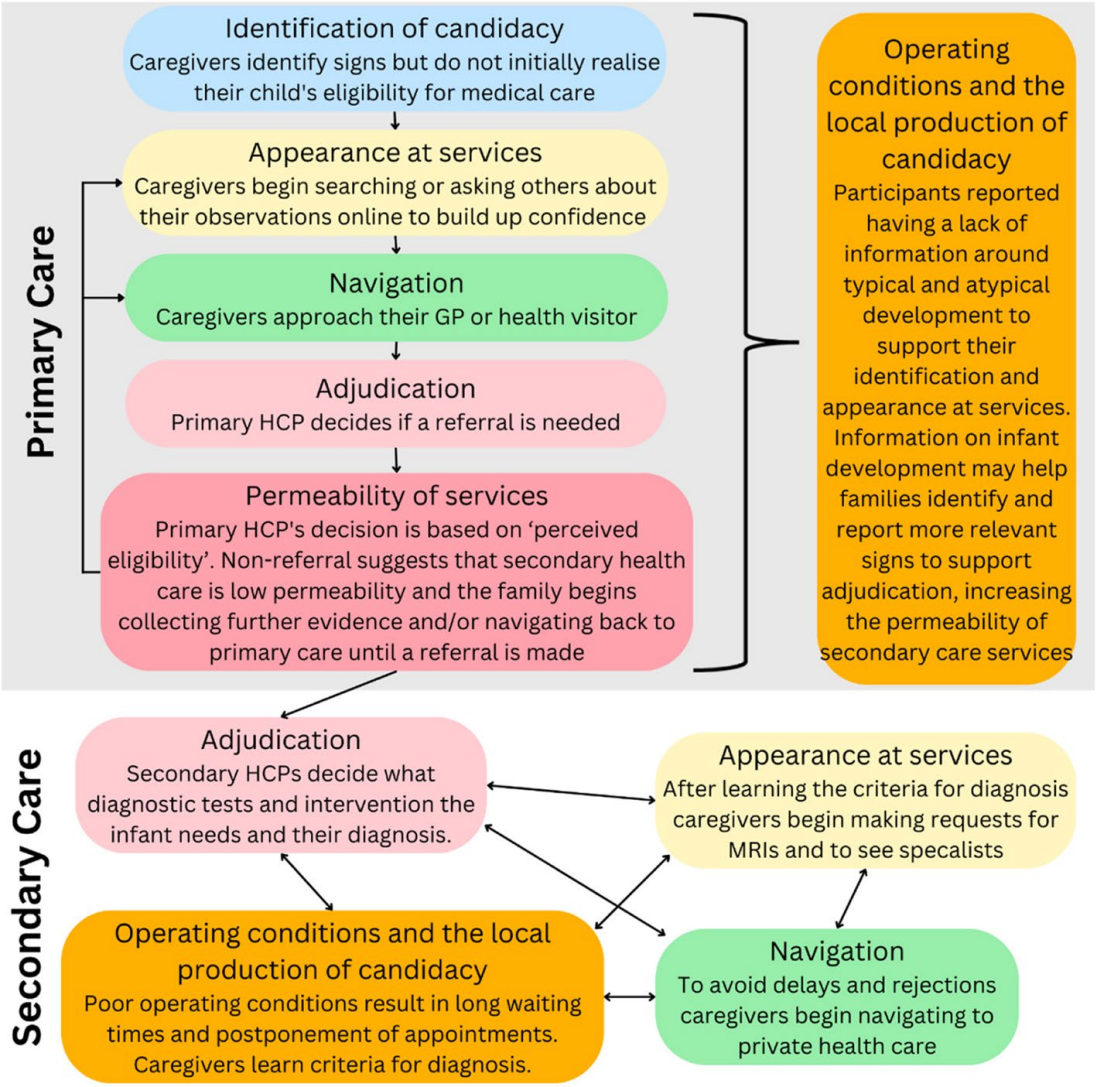
The order in which families of infants identified in the community with emerging motor problems and later diagnosed with CP go through the seven stages of candidacy. Candidacy is a dynamic process theory that describes how an individual negotiates their eligibility for health care with themselves and others. The dynamic nature of the theory means that the stages do not occur in a linear order, that two or more stages may occur at once, and that stages may reoccur. HCP – Health Care Professional. MRI – Magnetic Resonance Imaging brain scans

This Results section will now describe the evidence for each stage of candidacy in the order described by participants, shown in Fig. 1. It will then provide evidence for the type of information resources new families require and how an informational resource could support candidacy.

### Appraisal - identifying candidacy

Caregivers had difficulty initially identifying their child’s eligibility for health care resulting in delays. Even though they identified their infant’s initial symptoms, caregivers reported not seeking out medical attention straight away due to being ‘unaware’ that the symptom was not typical or because their infant seemed ‘fine’. Others thought they were ‘imagining’ the symptoms their infant was presenting, as outlined in the following quote:

At about 3–4 weeks we noticed a hand preference with movement. Being medical parents we downplayed this (!). By 8 weeks we were convinced. My Gran made a comment that he was “going to be a leftie” which made us realise that we were not imagining things. (F075)

In this case, a potential observation around ‘hand preference’ is initially downplayed by parents trying to compensate for their professional expertise and then confirmed over time through another observation matching their own.

One caregiver also reported a missed opportunity. A missed opportunity for referral occurred when both the caregiver and HCPs failed to recognise the child’s symptoms. This child did not receive referral until a teacher recognised the child’s symptoms. The child’s mother explained that he was her first child so she did not know that he was falling behind on development and the Health Visitor also did not notice anything unusual in his development. The mother (M167) reported her earliest concerns to be that her son was very stiff and needed help at age 3 years to play on climbing frames. By 5 years of age he was unable to jump and ‘constantly dribbled’. They (M167) reported that the first concerns raised to a HCP was that her son was unable to hold a pencil, unable to write, unable to balance, and was unable to judge depth at age 7 years. This child was 6+ years old when he received a diagnosis of mild (GMFCS II) Quadriplegia and was aged between 12–17 years at the time of the survey.

### Help-seeking

#### Appearance at services

Caregivers did not always know what their concerns were, with one parent describing how she had a ‘gut feeling’ something was wrong but could not explain what it was. Others felt they needed to build their confidence in their concerns before approaching a HCP. They described collecting more evidence before going to their General Practitioner (GP) by either talking to others, looking their concerns up on the internet, or by spending time further observing their infant’s symptoms. They described doing these things due to self-doubt making them think ‘it would be nothing and [that they would be] wasting an appointment’. (M033). For one caregiver the need to build confidence was due to the fear of being labelled as paranoid:

I remember pacing around the house holding the phone, dreading calling the GP for another appointment, for fear of being labelled the paranoid first time mother (despite being a midwife), but also knowing that something wasn't right and I HAD to call. (M039)

Some of these caregivers delayed seeking help after previously having their concerns dismissed by friends, family, or HCPs, leading them to doubt their own observations.

#### Navigation

Caregivers raised their concerns to health visitors or GPs. Some caregivers commented that they already had an appointment booked when they decided to seek help and so decided to wait rather than book an additional appointment.

### Diagnosis

#### Primary care adjudication and the permeability of services

Upon meeting with PHCP, the infants were either referred on immediately or were given a reason for not being referred on immediately (adjudication). The caregivers shared reasons for non-referral. The first occurred due to caregiver’s concerns being ‘brushed off’ by their GP or Health Visitor, or by the HCPs not sharing the caregiver’s concerns. The second was due to the PHCP offering an alternative reason for their infant’s symptoms, such as hypermobility or ‘late development’. The third was due to PHCPs choosing to ‘watch and wait’ for 2–3 months to see how the infant developed.. As such, these families often attended primary care before their infant met practitioner criteria for referral, suggesting the permeability for referral to be linked to age.

#### A split in the pathway

The adjudication by GPs resulted in families continuing different pathways. Those who did not receive a referral moved backwards along the pathway set out above. They began to search for more information: for some this meant spending ‘hours and hours [of] googling’ and ‘reading case studies’ so that they could present their findings to the GP. They continued to observe their infant’s development, and some continued to ask for advice from family and friends, all to improve their *appearance at services*.

Families who had initially approached health visitors *navigated* to GPs, whereas families who had initially approached a GP either revisited the same GP or approached a different GP in the hope of a different outcome. The caregivers described having attended primary care services up to five times before being referred.

#### Secondary care – the co-occurrence of service permeability, adjudication, and operating conditions resulting in local production of candidacy, navigation, and appearance at services

Once families received a referral, there were further barriers on the pathway to diagnosis. Some immediately faced problems with *permeability* and *adjudication*, as their GP referral was rejected by their local hospital. This family navigated back to their GP where they received a second referral to a different specialist. Other families were denied requests to be referred to other specialists or for an MRI brain scan.

In attending secondary care caregivers become more aware of the criteria needed for diagnosis. By becoming aware of the criteria, the caregivers changed their a*ppearance at health services*, as they began to request internal referrals to see specialists and for MRI brain scans. When their requests were denied or when they felt the waiting lists were too long, some caregivers *navigated* around these barriers by seeking out MRI brain scans from private companies. However, families only needed to *navigate* due to the poor *operating conditions* in secondary care. Additional examples of poor operating conditions include: postponement of appointments for ‘almost 6 months’ (M110), families being bounced ‘around the system’ between different hospitals and different specialists, and no cohesive communication between HCPs or between NHS Trusts.

#### Pre-treatment and offers and resistance to services

None of the participants described pre-treatment delays or reported rejecting a service offered to them by a health care professional.

### How to improve local production of candidacy: information for new families

Only interviewed participants were asked about the development of resources to aid in earlier identification. The parents of children with CP suggested information sheets would be better, rather than a questionnaire. This was because they experienced difficulties in finding information on typical and atypical development from a reliable source. In turn this made it difficult for them to determine whether their child’s development was atypical, reducing how credible their concerns were, impacting on their *appearance at services*. They felt that under the current system families need to take information with them to their GP, something that they would not be able to do with a questionnaire. This was in part due to their beliefs that GPs would benefit from further training on the early warning signs of CP, the ‘less obvious categories of CP’, and the different types of therapy services that can support infants.

The participants felt that providing new families with informational resources on infant development is ‘definitely something that that is needed’ (Bethany, IG1) and has ‘genuine value’ (Thomas, I15). The parents agreed that there was a need for multiple informational resources as they felt they knew ‘more or less nothing’ (Peter, I10) about infant development when they first brought their infant home. They suggested a short, to-the-point, hard copy resource that gives generic information on infant development related to key signs of CP, that would not overwhelm parents with too much information and would not scare them with the content. They also suggested a detailed online resource that new families could access for more information on typical and atypical development with signposting and clear safety advice in line with campaigns such as ICON cope (a preventative abusive head trauma campaign) [17]. Examples of the resources suggested by families are given in Table 5.

**Table 5:** Types of resources parents suggest could be used to improve identification of candidacy. Note: Short and long information resources refer to concise and detailed, respectively, information resources on early signs of CP. The personal child health record book (Red Book) is a national standard health and development record given to parents and carers after a child’s birth

***Short information resources***	
*Type of resource*	*Where it could be accessed*
A5 booklets, leaflets, parenting tips, posters in public spaces, and wallet card.	Baby groups, charities, the Healthy Start programme, Health visitor packs, parent classes, parenting groups, parenting websites, public spaces, personal child health record book, and wallet card.
***Long information resources***	
*Types of resource*	*Where it could be accessed*
Online digital resource	Hosted on a charities’ website, parenting website or other currently available online resource on child development

## Discussion

Overall, this study found that after noticing their infants first symptom, caregivers begin to look for information to determine whether they need to raise concerns about their infant. When they approach their HCPs, they may or may not be immediately referred to secondary care. Those who are not immediately referred begin searching for information again and continue returning to primary care services until a referral is made. Once referred, families face their referral being rejected, and poor operating conditions, which causes them to learn what is needed for diagnosis and navigate around it.

In this study families found access to information as the main limitation to self-produced candidacy, impacting on their on their appearances in services. The parents suggested the development of information resources may help new families find reliable information quicker, allow them to be more confident in raising their concerns and allow them to report all their infants’ signs rather than just their concerning observations. Parents asking for more information about infant development is not new. Both Cashin et al. [18] and Slomian et al. [19] asked new parents about what information they was most important for new parents to know. In both studies, parents rated information on infant development as essential.

With the delivery of health information there is always the concern for overwhelming primary care services with the worried well, however, there are several studies showing this not to be the case for information around infants. In 2016, Graybill et al. [20] gave new parents a 42-page Milestone Moments booklet on developmental milestones, that had been developed as part of the Centers for Disease Control and Prevention’s National Centre on Birth Defects and Developmental Disabilities (CDC-NCBDDD) Learn the Signs/Act Early (LTSAE) initiative. They found that the booklets significantly improved parental knowledge around general child development. Although the booklets helped parents to identify concerns about their child’s development, they did not change the rates of parent-initiated referral. Similarly, in 2016 the ‘little orange book’ was published by the Newcastle Gateshead Clinical Commissioning Group and trialled in the Northeast of England to see if it could reduce unnecessary attendance at emergency services [21]. The little orange book contains information about the different illnesses likely to affect 0–5-year-olds and informs parents on how to self-manage childhood illnesses or signposts them to the service that can provide adequate support. An evaluation survey demonstrated that families found the book credible due to its association with the NHS as well as an extremely important tool to aid decision making. Additionally, the evaluation found that 57% of participants felt the information made them more likely to attend non-emergency services instead of emergency services. As such, both studies demonstrate parental education as key tool to helping parents identify whether their child requires medical attention.

Further research is needed to develop educational resources for new parents around infant development in the UK. Our research showed there is a lack of consensus on the best way to provide the materials and the ideal scope of content. The CDC-NCBDDD have already began developing an online milestone checklist for children aged 0–5 years [22]. Although this is encouraging, it does not cover the earlier signs of CP that new parents may identify, such as early hand preference and tone, or the signposting that is relevant to the NHS pathways.

The journeys described by participants match closely to those described for other paediatric conditions, such as cancer and diabetes [23–26]. It is likely that candidacy is an underlying factor of relevance across primary care. Further research is needed to understand if access to information is a barrier across all paediatric conditions or if other aspects of candidacy are influencing patient journeys.

This study focused on the information provision due to it being one of the more prevalent barriers described by participants and being relatively easy to fix. In 2014, a Parliamentary inquiry [27] recommended the following to improve earlier identification of CP signs: a greater emphasis on parental concerns, commitment to rapid referral and elimination of watch and wait approaches, more widespread use of Prechtl’s general movements assessment, and improving awareness of CP among GPs and Health Visitors. However, apart from the use of Prechtl’s general movements assessment, these recommendations were not included in the NICE guidelines on CP in under 25’s [28]. Shortly after, the Richardson [29] report provided two suggestions of approaches used by the CP Alliance in Australia which could be implemented in the UK.

The first approach suggested was a CP register. The Australian CP register provides a list of infants identified as being at risk of CP in hospital and within the community, and enters these infants into an adjoining screening program, CP Check-Up, described below. The CP register includes a community advisory team who provide support to PHCPs with identification of infants within the community.

The second approach was the implementation of screening programs. Richardson [29] described 4 overlapping screening programs. 1) Neonates, for 0–3 month high risk infants. 2) 3 month assessment, consisting of the GMs, the Hammersmith Infant Neurological Assessment and the Bayley Scales of Infant and Toddler Development assessments. 3) Early Detection and Diagnosis clinics, a follow up service for neonates, also accepting referrals from parents, GPs, community therapists and paediatricians. 4) CP check-up, a comprehensive and holistic surveillance program for infants at risk of, or diagnosed with, CP. Infants in this program receive assessments every six months between birth and 6 years, and yearly appointments after 6 years.

Together, the Australian CP register and CP screening programs resulted in around 50% of infants attending the CP Alliance clinics being diagnosed within the first year of life, 75% by their second year, and 90% by their third.

In relation to all types of referrals in primary care, Greenwood-Lee et al. [30] suggest that guideline and educational interventions for PHCPs should be built on by incorporating communication with secondary care specialists, such as: referral reply letters from Secondary Health Care Professionals (SHCPs); relationship building and collaboration on care practices between PHCPs and SHCPs; peer review and/or supported patient assessment implemented through primary triage clinics within secondary care; and peer review groups between PHCPs with consultant engagement. Alternatively, Greenwood-Lee et al. [30] also suggested the implementation of standardised referral forms, checklists, scoring systems, and assessment tools specifically designed to be used within primary care to help improve referral quality and decrease delayed referrals and unnecessary referrals. These suggestions are supported by Blank et al.’s [31] systematic review of problems and solutions in primary care referral. Although Greenwood-Lee et al. (2018) and Blank et al. (2014) do not directly support Richardson’s [29] proposal, they do agree that a broader, richer, referral infrastructure is needed, which increases the level of skills within primary care.

CP registers and UK screening programs have previously been available in the UK. In the 2014 Parliamentary Inquiry [27], the UK charity SCOPE provided evidence of an advisory assessment service (AAS) they had previously provided in London that was accessible to families across the UK. The AAS gave parents access to a 2–3 day assessment carried out by a multi-disciplinary team of professionals. The team would provide the parents with a detailed report of the infant’s specific needs and provide signposting to appropriate follow-on services. The service was offered as evidence as a way to improve CP identification, however required funding to be able to restart. Since the Inquiry, this service has not been restarted. Similarly, the provision of a CP register was also recommended, however, at the time of writing, a UK wide CP register has not been created despite registers already existing in Northern Ireland and Scotland. Thus, despite suggestions being put forward on how to improve the UK CP screening program, none of these suggestions have been put into action.

The significant challenges facing primary HCPs in identifying infants with emerging CP amongst the far greater number with non-specific developmental and behavioural concerns should not be underestimated and deserve further exploration in the interests of promoting timely intervention for affected infants and their families. To help reduce delays around early identification and referral primary HCPs should refresh and update their knowledge on the early signs of CP [32].

The limitations of the study should be discussed. Firstly, both samples were over-representative of White European women of higher socio-economic status (SES). Every year the GP patient survey is conducted within the UK to allow patients to feed back about their experiences and the services they have received [33]. The GP patient survey has continually shown patients from any Asian background, any mixed background, and any other ethnic group to report more ‘poor’ experiences with their GP practice than those from white or black/African/Caribbean backgrounds [34]. Studies using the GP patient survey data [34, 35] have shown ethnic minorities are significantly less able to get an appointment on the same day or within 2 days of asking or to get an appointment with a particular GP, compared to white patients. Additionally, they are significantly less satisfied with their GPs opening hours and being able to get through to their GP surgery on the phone. PHCPs have also reported that patients from minority ethnic backgrounds can have different cultural expectations and understandings of the UK health care system and that they may have language difficulties which act as further barriers to them accessing medical care [36].

Risk of CP is negatively associated with SES, even when confounding variables, such as multiple births, are controlled for [37]. Lower SES is associated with lower income, lower education, poor housing, increased health care needs, and increased barriers to research participation, such as feeling unqualified to take part and requirement for additional carer time to aid participation [38]. It is likely that informational tools may not be what all families want or need and such tools may not be accessible to those with lower literacy abilities. Additionally, the experiences shared may not reflect the difficulties that those from lower SES face when trying to seek care for their infant. Individuals from lower SES often experience more barriers to participating in research. As participants were informed that the interview would last around an hour, some may not have been able to guarantee that time due to work schedules and childcare, which are two barriers known to impact those from low SES [38].

The modal age for the children reported about was 6–11 years, with 22% of the sample responding about a child aged over 12 years. Primary care practices have changed over the last two decades, including the development and implementation of care pathways and treatment guidelines [28, 39]. As such, the responses may not be reflective of the current process. By not having a proportional representation of the current UK population this study may not fully represent the first concerns parents develop in the UK.

Additionally, none of the participants reported denying offers of support. Caregivers have various coping strategies towards accepting that their child is not developing typically, including avoidant coping styles [40]. Targeted interviews with HCPs may allow for exploration of diagnostic journeys of families who are unlikely to take part in research.

The survey and interviews were retrospective and therefore may be subject to recall and response bias. As the findings were not checked against medical records, information provided could not be verified.

## Conclusions

Parents face difficulties in identifying their infant’s eligibility for medical care which has effects on how they present in primary care and the decisions made by PHCPs. When PHCPs decide not to refer on when families first raise their concerns, families can begin to repeat the process of finding information to support their infant’s eligibility and reappearing within primary care services. Once a referral is made, families may face multiple barriers in determining their child’s eligibility, including poor operating conditions and in rare cases, a reported reluctance on the part of HCPs to refer them promptly for investigations required for diagnosis. As a result parents and caregivers begin to identify their needs and navigate through the system, such as attending private care appointments, to decrease the time to diagnosis. Additional parental education on infant development is one way that parents find acceptable that could help parents raise their concerns earlier with more thorough awareness of their infants’ difficulties. PHCPs are also open to the development of informational resources to help them identify infants demonstrating early movement difficulties. However, informational interventions alone are not enough to solve the barriers faced by families in the CP diagnosis journey. Further research is needed to understand how information more specific to CP impacts parental decision making, and to optimise the CP diagnosis and referral pathway.

## Data Availability

The datasets generated and/or analysed during the current study available from the corresponding author on reasonable request.

## Abbreviations

CDC-NCBDDD: Centers for Disease Control and Prevention’s National Centre on Birth Defects and Developmental Disabilities
CP: Cerebral Palsy
GMFCS: Gross Motor Function Classification System
GP: General Practitioner
HCP: Health Care professional
MRI: Magnetic Resonance Imaging
PHCP: Primary Health Care Professional
SES: Social Economic Status
SHCP: Secondary Health Care Professional
